# Simple gravitational particle swarm algorithm for multimodal optimization problems

**DOI:** 10.1371/journal.pone.0248470

**Published:** 2021-03-18

**Authors:** Yoshikazu Yamanaka, Katsutoshi Yoshida

**Affiliations:** Department of Mechanical and Intelligent Engineering, Utsunomiya University, Utsunomiya, Tochigi, Japan; Torrens University Australia, AUSTRALIA

## Abstract

In real world situations, decision makers prefer to have multiple optimal solutions before making a final decision. Aiming to help the decision makers even if they are non-experts in optimization algorithms, this study proposes a new and simple multimodal optimization (MMO) algorithm called the gravitational particle swarm algorithm (GPSA). Our GPSA is developed based on the concept of “particle clustering in the absence of clustering procedures”. Specifically, it simply replaces the global feedback term in classical particle swarm optimization (PSO) with an inverse-square gravitational force term between the particles. The gravitational force mutually attracts and repels the particles, enabling them to autonomously and dynamically generate sub-swarms in the absence of algorithmic clustering procedures. Most of the sub-swarms gather at the nearby global optima, but a small number of particles reach the distant optima. The niching behavior of our GPSA was tested first on simple MMO problems, and then on twenty MMO benchmark functions. The performance indices (peak ratio and success rate) of our GPSA were compared with those of existing niching PSOs (ring-topology PSO and fitness Euclidean-distance ratio PSO). The basic performance of our GPSA was comparable to that of the existing methods. Furthermore, an improved GPSA with a dynamic parameter delivered significantly superior results to the existing methods on at least 60% of the tested benchmark functions.

## Introduction

Multimodal optimization (MMO) algorithms [1] (also known as niching methods or techniques) can locate multiple global optima in a single run, which is essential for solving many scientific and engineering optimization problems, e.g., Toyota paradox [2], motor design [3], clustering validity functions [4], network modeling [5], truss-structure design [6], overlay network [7], multi-robot cooperation [8], wireless sensor network [9], object detection [10], and honeycomb core design [11]. Compared with unimodal optimization (UMO) algorithms that can locate just a single global optimum, MMO algorithms have several benefits in some real-world problems [1], where some factors can be difficult to model mathematically, e.g., degree of difficulty in manufacturing. Specifically, having multiple solutions with a similar quality will give a decision maker more options for consideration, with factors that are not captured in the mathematical model. Finding multiple solutions may also help to reveal hidden properties or relations of the problem, e.g., the distribution of the solution set in the problem space. Therefore, MMO algorithms provide richer information about the problem domain than the UMO algorithms.

To achieve the MMO algorithms, several well-known niching techniques have been developed since the 1970s, namely, crowding [12], deterministic crowding [13], fitness sharing [14], derating [15], restricted tournament selection [16], clustering [17], and speciation [18]. Initially, these niching techniques were developed for evolutionary algorithms (EAs) and genetic algorithms (GAs).

Since the 2000s, some MMO algorithms based on particle swarm optimization (PSO) [19–21] have been proposed [22–24]. These algorithms simply replace the global best of classic PSO with neighborhood best; hence, they can be categorized into a one-stage method like the classic PSO. Owing to their simplicity, they have been successfully applied to many real-world problems [4–11]. However, these one-stage MMO algorithms are known to perform poorly compared with state-of-the-art niching methods [24, 25].

Recently, two-stage MMO algorithms [26–29] won the multimodal optimization competitions held by the Congress on Evolutionary Computation (CEC) and the Genetic and Evolutionary Computation Conference (GECCO). The first stage of the two-stage niching separates the candidate solutions into subpopulations by a clustering algorithm [28]. In the second stage, each subpopulation seeks the optimum by a core algorithm, which utilizes a restart scheme and taboo archive. However, non-experts in optimization algorithms hesitate to apply such complicated algorithms to their real-world problems. Although the source codes of these algorithms are available, they may not be directly implementable in the production items of a firm. In such situations, engineers should understand and implement the algorithms by themselves [30]. Therefore, it seems that the existing MMO algorithms have a dilemma between searching performance and algorithmic complexity.

The present study aims to develop a simple and powerful algorithm, which can be easily understood and implemented even by non-experts in optimization algorithms and can outperform the existing one-stage methods. The present paper proposes a new, simple, and purely dynamical one-stage method called the gravitational particle swarm algorithm (GPSA). This method replaces the linear feedback term involving the global best in the classical PSO framework with the inverse-square gravitational force between the particles. Under this mechanism, sub-swarms will autonomously self-organize at the nearby optima, but a few particles will intermittently escape, thereby maximizing the coverage of widely distributed multiple optima.

As our GPSA automatically and dynamically generates the above-mentioned swarm behavior without any clustering algorithms, restart scheme, and taboo archive, it is tractable even for non-experts. Furthermore, it requires fewer computational resources and fewer tuning parameters than existing MMO algorithms [22–24, 26, 31, 32]. On the CEC 2013 niching test problems [33], our GPSA performed comparably to the existing methods [22, 25], and can significantly outperform them by assigning a dynamic parameter.

Our GPSA differs from the existing one-stage algorithms based on PSO [22–24]. In particular, our GPSA omits the algorithmic particle-selecting procedures—sorting and selecting personal-best or nearest best(s)—of these algorithms. It also differs from ring-topology PSO (RPSO) [25] because it does not use ring-topology. Furthermore, our GPSA differs from methods based on gravity force [34–36] that cannot detect multiple global optima. Although a gravity-force method that detects multiple global optima has been proposed [32], this method requires algorithmic particle-selecting procedures, which are omitted in our GPSA. In addition, other well-known niching techniques utilized in EAs and GAs [12–18] are not required in our GPSA.

The remainder of this paper is organized as follows. Section 2 discusses the MMO problems and the drawbacks of the existing methods [22–29, 31, 32]. Section 3 introduces our GPSA, describes its search mechanism, and demonstrates its niching capability on one- and two-dimensional functions. Section 4 evaluates the performance of our GPSA on the CEC 2013 niching test problems, and confirms the comparable performances of our GPSA and the existing one-stage methods. In Section 5, our GPSA is improved by assigning a dynamic parameter. The superiority of the improved version over the existing methods is demonstrated in this section. Section 6 concludes the paper.

## Background and related work

### MMO problems

Consider an optimization problem of the form

$$\begin{array}{c} \underset{x}{\text{Maximize}}f\left(x\right),\;x\in R^{d}, \end{array}$$ (1)

where ***x*** is a *d*-dimensional vector and $f:R^{d}\to R$ is a real-valued function. This study focuses on the case where multiple global optima satisfy (1), i.e.,

$$\begin{array}{c} x_{k}^{*}=\text{arg}\;\text{max}\;f(x),\;k=1,2,\cdots ,N^{\text{go}}, \end{array}$$ (2)

where $x_{k}^{*}$ is *k*th global optima and *N*^go^ is the number of these global optima. Such problems are said to be multimodal, namely, they are MMO problems [1]. Conversely, problems with a single global optimum (*N*^go^ = 1) are known as UMO problems.

Eq (1) can be solved by particle-swarm-based approaches by considering the motion of a swarm of *N* candidate solutions:

$$\begin{array}{c} X\left(t\right)≔\left\{x_{1}\left(t\right),x_{2}\left(t\right),\ldots ,x_{N}\left(t\right)\right\},\;x_{i}\left(t\right)\in R^{d},\;t=0,1,\cdots ,t_{\max}, \end{array}$$ (3)

where *t* is discrete time. The candidate solutions (called particles) explore the *d*-dimensional domain $R^{d}$, seeking either multiple global optima (in MMO problems) or a unique optimum (in UMO problems). The performances of such approaches therefore depend on the swarm movements.

### Classical PSO

PSO was originally proposed in [19] and [20], and numerous variants for improvements have been proposed. In a typical classical PSO [21], the motion of the *i*th particle *x*_*i*_(*t*) is recursively described as [37]:

$$\{\begin{array}{ll} v_{i}(t+1)=\omega v_{i}(t)+c_{1}r_{1i}(t)⊗(pb_{i}(t)-x_{i}(t))+c_{2}r_{2i}(t)⊗a_{i}(t), & \left(4\text{a}\right)\\ x_{i}(t+1)=v_{i}(t+1)+x_{i}(t),\;t=0,1,\ldots ,t_{\text{max}}, & \left(4\text{b}\right) \end{array}$$

with

$$\begin{array}{c} a_{i}\left(t\right)=gb\left(t\right)-x_{i}\left(t\right), \end{array}$$ (5)

where ⊗ denotes component-wise multiplication, ***v***_*i*_(*t*) is the velocity of the *i*th particle, and ***r***_1*i*_(*t*) and ***r***_2*i*_(*t*) are independent white random-process vectors whose components are uniformly distributed over [0, 1]. The personal bests, denoted by ${pb}_{i}\left(t\right)\in R^{d}$, are the highest-cost positions found by each particle thus far (up to time *t*) among ***x***_*i*_(0), ⋯, ***x***_*i*_(*t*). The global best $gb\left(t\right)\in R^{d}$ is the best solution found by any particle up to time *t*. $\left(\omega ,c_{1},c_{2}\right)\in R^{3}$ are tuning parameters.

Eqs (4) and (5) imply that only single solutions are found, as the global feedback term (5) obtains a unique limit. This problem has already been demonstrated by [25].

### Two-stage MMO algorithms

To overcome the problem of classical PSO, researches have proposed two extended PSO methods: niching migratory multi-swarm optimizer (NMMSO) [26] and multi-charged particle swarm optimization (mCPSO) [31]. These methods, called two-stage niching methods [28], separate the particles into sub-swarms by a clustering algorithm in the first stage, and perform sub-swarming searches of the optimum by the classical PSO in the second stage. Alternative two-stage methods, which utilize an evolutionary strategy in the second stage, include covariance matrix self-adaptation with repelling subpopulations (RS-CMSA) [27], the Hill-Valley evolutionary algorithm (HillVallEA) [28], and its improved variant (HillVallEA19) [29]. These two-stage methods also utilize additional sub-procedures, namely, a restart scheme in NMMSO, mCPSO, RS-CMSA and HillVallEA and a taboo archive in RS-CMSA.

Unfortunately, the complexity of these two-stage methods is daunting to engineers wishing to implement search algorithms in their production items. Although the source codes of these methods are available, they might not be directly implementable in the production items of a firm. In such situations, engineers should understand and implement the algorithms by themselves, as reported in [30].

### One-stage MMO algorithms

Simpler niching methods than the two-stage methods are also available. Some of these algorithms are based on PSO; specifically, RPSO, fitness Euclidean-distance ratio PSO (FERPSO) [22], locally informed particle swarm (LIPS) [24], and species-based PSO (SPSO) [23]. Another niching method, called the niching gravitational search algorithm (NGSA) [32] uses gravitational forces. In the present study, these methods are called one-stage methods because they include no clustering algorithms. Table 1 summarizes the characteristics (computational complexity, number of tuning parameters, and algorithm type) of these one-stage methods.

**Table 1:** Characteristics of the existing one-stage niching methods.

	RPSO	FERPSO	LIPS	SPSO	NGSA
Complexity:					
*O*(*N*) ^a^	✔	n/a	n/a	n/a	n/a
*O*(*N*^2^ + *α*) ^b^	n/a	✔	✔	✔	✔
The number of Parameters:					
	3	3	4	4	5
Type:					
Based on PSO	✔	✔	✔	✔	n/a
Using gravitational force	n/a	n/a	n/a	n/a	✔

“✔” and “n/a” indicate applicable and not applicable, respectively.

^a^the same as classical PSO

^b^obtained by calculating the Euclidean norm between the particles and by selecting or sorting of particles, where *α* = *N* or *N* log *N*. *N* is the number of particles.

Focusing on the computational complexity, these existing methods can be broadly classified into two groups: RPSO with complexity *O*(*N*); and FERPSO, LIPS, SPSO and NGSA with complexity *O*(*N*^2^ + *α*), where *α* = *N*, *N* log *N*. In the method of the former group (RPSO), the complexity is equivalent to that of the classical PSO (4). In the methods of the latter group, which calculate the Euclidean norm between particles, the complexity is required to be at least *O*(*N*^2^). In addition, FERPSO adds a complexity *O*(*N*) for particle selection, whereas LIPS, SPSO and NGSA add a complexity *O*(*N* log *N*) for particle sorting.

With the exception of RPSO and FERPSO, the number of tuning parameters is one or two higher in these methods than in the classical PSO. The additional parameters are required for sorting the particles.

The comparison confirms RPSO as the simplest niching method, but RPSO is known to deliver poorer performance than the other methods [25].

The above discussion highlights the drawbacks of the existing high-performance niching methods, namely, the difficulties in understanding and implementing two-stage methods, and the high computational complexity and large number of tuning parameters in one-stage methods.

## GPSA

To resolve the above drawbacks, the present study proposes a new and simple niching method called GPSA. The basic idea is that, if the particles in PSO attract each other, they can autonomously organize into sub-swarms and search the multiple global optimum without any additional procedures. Specifically, the classical global feedback term (5) is replaced with a term that introduces inverse-square gravitational forces between the particles, i.e.,

$$\begin{array}{c} a_{i}\left(t\right)=\sum_{k\neq i}\frac{1}{\left|\right|d_{ki}\left(t\right){\left|\right|}^{2}}u_{ki}\left(t\right), \end{array}$$ (6)

$$\begin{array}{c} u_{ki}\left(t\right)=\frac{d_{ki}\left(t\right)}{\left|\right|d_{ki}\left(t\right)\left|\right|},\;d_{ki}\left(t\right)=x_{k}\left(t\right)-x_{i}\left(t\right), \end{array}$$ (7)

where ||⋅|| denotes the Euclidean norm, ***d***_*ki*_(*t*) is a displacement vector between the positions of the *i*th and *k*th particles, and ***u***_*ki*_(*t*) is the normalized vector of ***d***_*ki*_(*t*). Algorithm 1 shows the pseudocode of our GPSA.

The novelty of our GPSA is summarized below.

- Our method is a purely dynamical one-stage method, i.e., the particles are updated only by a dynamical system with no additional procedures. In contrast, the existing two-stage methods require a clustering algorithm, a restart scheme, or a taboo archive [26–29, 31].
- Unlike the existing one-stage methods [22–24, 32], our method requires no algorithmic procedure for selecting the social or nearest best(s).

**Algorithm 1** GPSA

 **Input** *f*, *d*, *N*, *t*_max_, *ω*, *c*_1_, *c*_2_

 **Output** The set of final personal bests {***pb***_1_(*t*_max_), ***pb***_2_(*t*_max_), …, ***pb***_*N*_(*t*_max_)}

1: **procedure** GPSA

2:  *t* = 0

3:  Initialize particles’ position $x_{i}\left(t\right)\in R^{d},i=1,2,\ldots ,N$

4:  Initialize particles’ velocity $v_{i}\left(t\right)\in R^{d}$

5:  Set personal bests via ***pb***_*i*_(*t*) = ***x***_*i*_(*t*)

6:  **while** *t* < *t*_max_ **do**

7:   **for** *i* = 1, …, *N* **do**

8:    Update particles’ position and velocity via (4) with (6)

9:    **if** *f*(***x***_*i*_(*t* + 1)) > *f*(***pb***_*i*_(*t*)) **then**    ▹ Update personal best

10:     ***pb***_*i*_(*t* + 1) = ***x***_*i*_(*t* + 1)

11:    **else**

12:     ***pb***_*i*_(*t* + 1) = ***pb***_*i*_(*t*)

13:   *t* = *t* + 1

Therefore, our GPSA is advantaged by simplicity. Our GPSA can be easily understood and implemented by non-experts in optimization algorithms.

**Fig 1:**
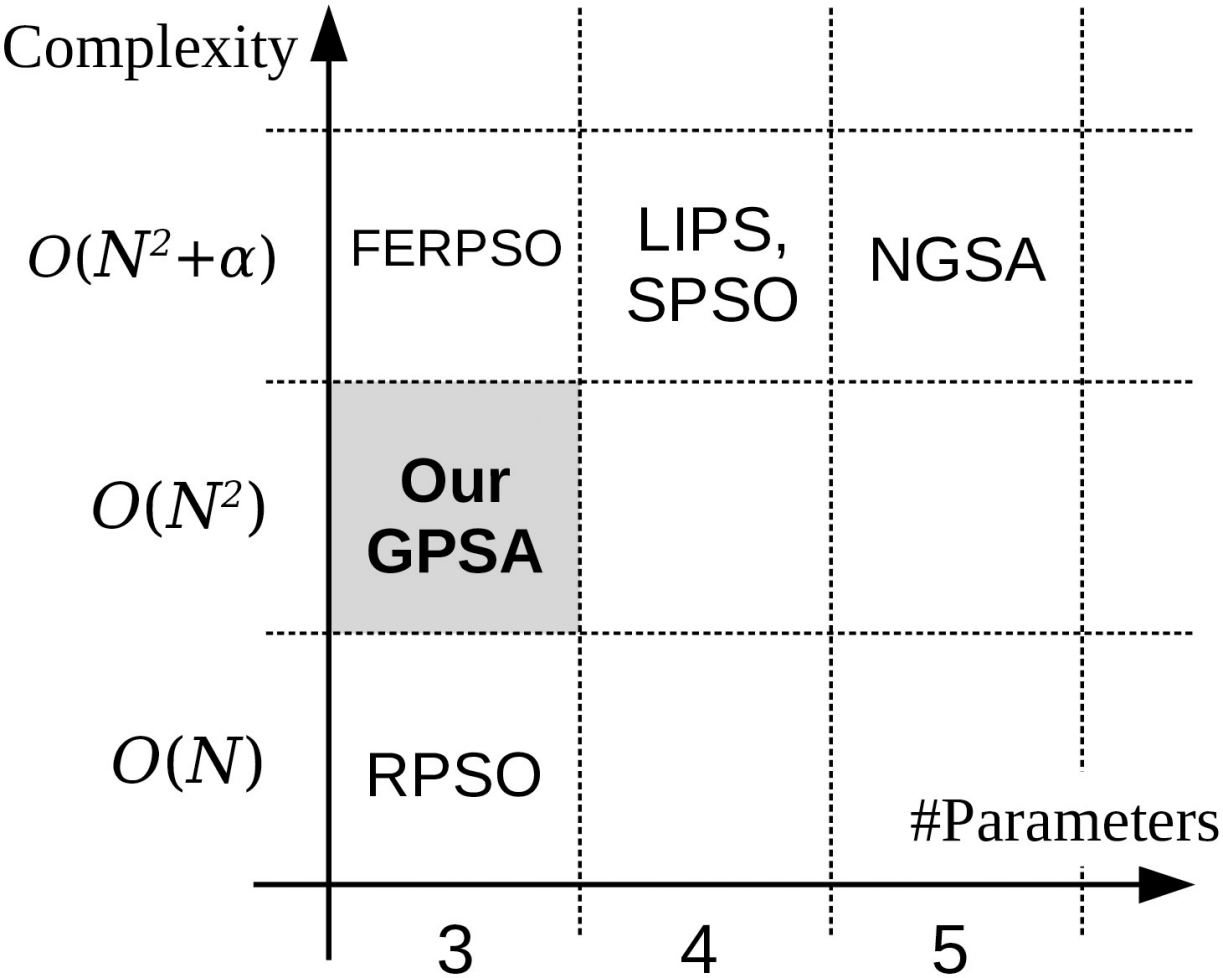
Comparison of the characteristics (computational complexity and number of tuning parameters, #parameters) of our proposed GPSA and existing one-stage niching methods.

Fig 1 compares the characteristics (computational complexity and number of tuning parameters) of our GPSA with those of the existing one-stage methods. As evidenced in the figure, our GPSA has the same number of tuning parameters $\left(\omega ,c_{1},c_{2}\right)\in R^{3}$ as RPSO and FERPSO, and fewer parameters than other one-stage methods. In addition, the complexity of our GPSA is *O*(*N*^2^), obtained by summing the complexities *O*(*N*(*N* − 1)) of (6) and *O*(*N*) of (4). This complexity is higher than in the *O*(*N*) method, but lower than in the *O*(*N*^2^ + *α*) methods, where *α* = *N* or *N* log *N*. Therefore, in terms of the number of tuning parameters and complexity, our GPSA is simpler than these existing one-stage methods except RPSO.

Although the nominal performance of our GPSA is apparently inferior to that of RPSO in Fig 1, the actual performances of GPSA and RPSO are comparable. Furthermore, after minor improvements, our GPSA significantly outperforms RPSO. This performance will be demonstrated after the following examples.

### One-dimensional examples

Our proposed GPSA dynamically organizes the niching behavior of the sub-swarms. This mechanism is clarified through illustrative examples in the present and following sub-sections.

**Fig 2:**
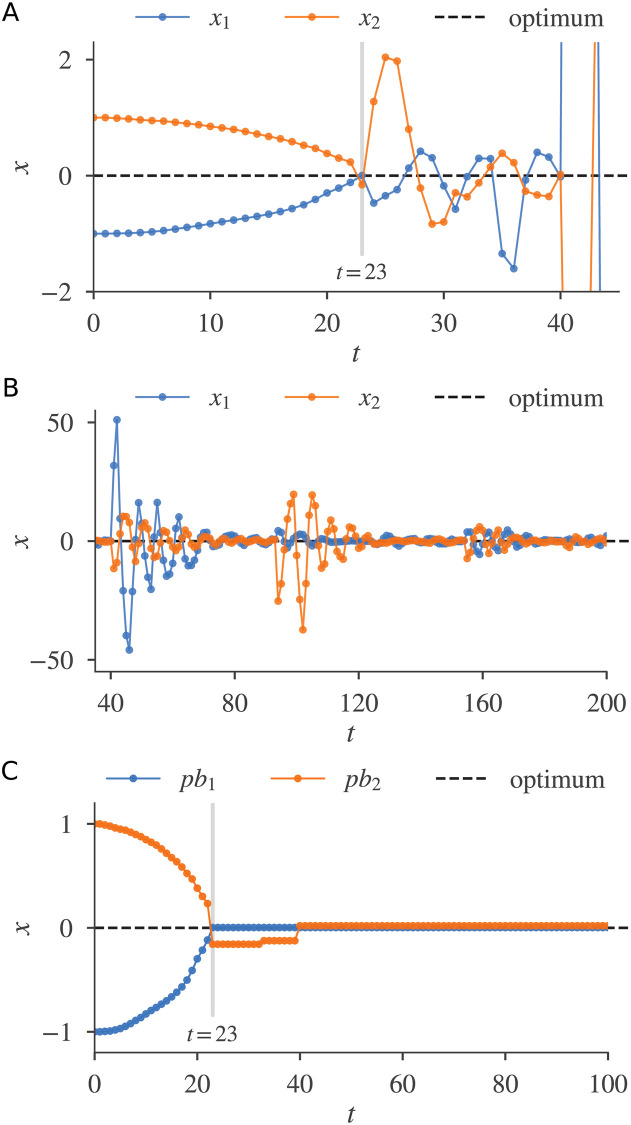
A two-particle GPSA solving a one-dimensional UMO function. (A) Particle positions (0 ≤ *t* ≤ 45). (B) Particle positions (35 ≤ *t* ≤ 200). (C) Personal bests.

Fig 2 shows the motions produced by a two-particle GPSA solving a one-dimensional UMO problem, *f*(*x*) = −*x*^2^ (an inverted sphere). Here the GPSA parameters were set to *ω* = 0.729 and *c*_1_ = 1.49445. These values are known to prevent particle divergence in the classical PSO [38], and are used even in the existing niching PSOs [22, 25]. The newly introduced parameter *c*_2_ was set to 0.05.

During the initial phase (0 ≤ *t* ≤ 23), the particles were mutually attracted by gravity. At some close enough distance (*t* = 23), they appeared to repel one another. Such particle repulsion behavior, called a repulsive flip here, is considered as a gravity-induced motion. Roughly speaking, ignoring the randomness and setting *ω* = *c*_1_ = 0 in (4a), the one-dimensional gravitational force (6) becomes

$$\begin{array}{c} a\left(t\right)≔a_{1}\left(t\right)=-a_{2}\left(t\right)=\frac{x_{2}\left(t\right)-x_{1}\left(t\right)}{d{\left(t\right)}^{3}},\;d\left(t\right)=\left|x_{2}\left(t\right)-x_{1}\left(t\right)\right|, \end{array}$$ (8)

so the positions of the two close particles are updated as

$$\begin{array}{c} x_{1}\left(t+1\right)=x_{1}\left(t\right)+c_{2}a\left(t\right),\;x_{2}\left(t+1\right)=x_{2}\left(t\right)-c_{2}a\left(t\right). \end{array}$$ (9)

If $d(t)=\sqrt[{3}]{c_{2}}$, the particles are swapped as

$$\begin{array}{c} x_{1}\left(t+1\right)=x_{1}\left(t\right)+c_{2}a\left(t\right)=x_{1}\left(t\right)+c_{2}\frac{x_{2}\left(t\right)-x_{1}\left(t\right)}{c_{2}}=x_{2}\left(t\right). \end{array}$$ (10)

**Fig 3:**
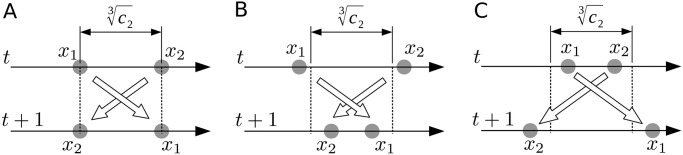
Different gravity-induced behaviors of two nearby particles. (A) Swap. (B) Attractive flip. (C) Repulsive flip.

Similarly, we obtain

$$\begin{array}{c} x_{2}\left(t+1\right)=x_{1}\left(t\right), \end{array}$$ (11)

as shown in Fig 3(A). In addition, when

$$\begin{array}{c} d\left(t\right)=\sqrt[{3}]{c_{2}}+\epsilon_{+},\;0<\epsilon_{+}\ll \sqrt[{3}]{c_{2}}, \end{array}$$ (12)

there is what we call an attractive flip (Fig 3(B)), because

$$\begin{array}{c} d\left(t+1\right)=|x_{2}\left(t+1\right)-x_{1}\left(t+1\right)|=\left|x_{2}\left(t\right)-x_{1}\left(t\right)-2c_{2}a\left(t\right)\right|=|\frac{d{\left(t\right)}^{3}-2c_{2}}{d{\left(t\right)}^{2}}|<\sqrt[{3}]{c_{2}}. \end{array}$$ (13)

In contrast,

$$\begin{array}{c} d\left(t\right)=\sqrt[{3}]{c_{2}}-\epsilon_{+} \end{array}$$ (14)

generates a repulsive flip because $d(t+1)>\sqrt[{3}]{c_{2}}$ (Fig 3(C)). From (13), we established that $d\left(t+1\right)=|\frac{d{\left(t\right)}^{3}-2c_{2}}{d{\left(t\right)}^{2}}|\to \infty \;\left(d\left(t\right)\to 0\right)$. In other words, the repulsive flip strongthens as *d*(*t*) approaches 0.

After the repulsive flip at *t* = 23 (Fig 2(A)), each particle underwent different damped oscillations around *pb*_*i*_(*t*), because the second term in (4a) imposes a linear restoring force. After the second repulsive flip at *t* = 40, repeated mutual repulsive flips of the particles were followed by individual dumped oscillations (Fig 2(B)). During this time, the particle’s personal bests, *pb*_1_(*t*) and *pb*_2_(*t*), rapidly converged to the problem optimum *x* = 0 (Fig 2(C)), confirming that our GPSA can solve the UMO problem.

**Fig 4:**
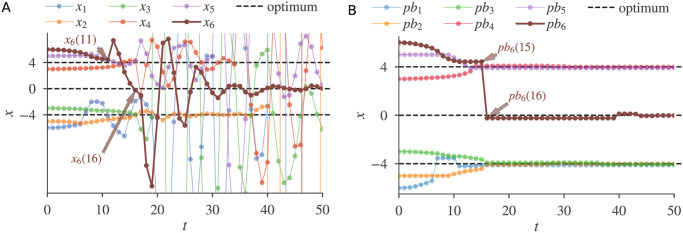
A six-particle GPSA solving a one-dimensional MMO function with three global optima. (A) Particle positions. (B) Personal bests.

The search motions of our GPSA also effectively solve MMO problems. Fig 4 shows the motions of six particles searching for the three global optima of the function *f*(*x*) = max{−|*x* − 4|^2^, −|*x*|^2^, −|*x* + 4|^2^}. The particles successfully found all three solutions. Initially, our GPSA autonomously and dynamically generated multiple sub-swarms near two of the optima (Fig 4(A)). A repulsive flip between *x*_5_(*t*) and *x*_6_(*t*) at *t* = 11 then repelled *x*_6_(*t*), which underwent a large-amplitude damped oscillation. This repulsion and oscillation process helped the personal best *pb*_6_(*t*) to find the distant optimum *x* = 0 (Fig 4(B)).

### Two-dimensional example

**Fig 5:**
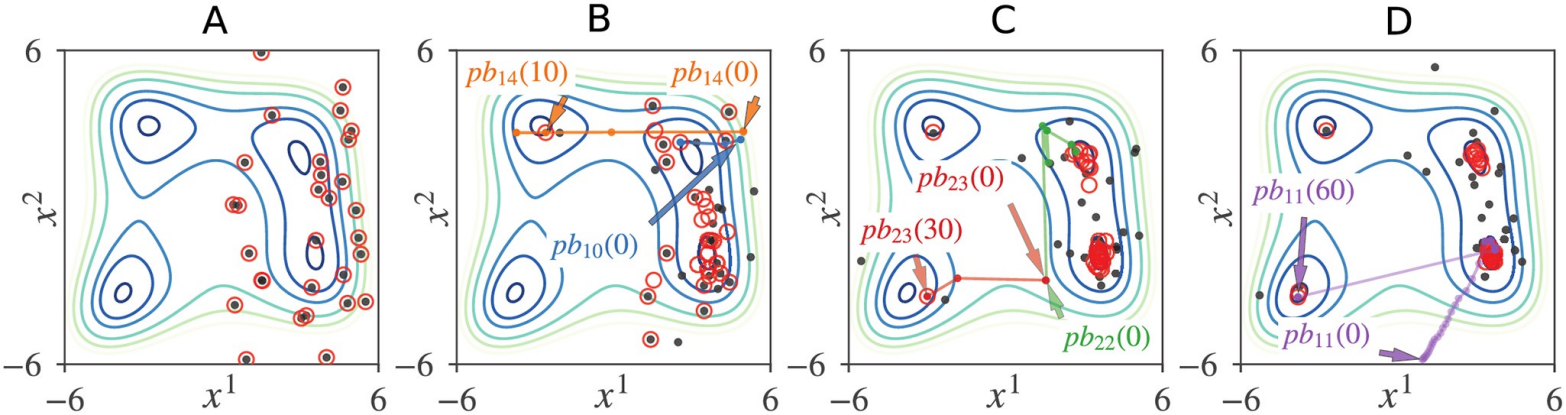
Snapshots of a GPSA run that found all four global optima of the Himmelblau function. *x*^*j*^ denotes the *j*th axis of position vector *x*, and the black and red circles indicate the positions and personal bests of particles, respectively. (A) *t* = 0. (B) *t* = 10. (C) *t* = 30. (D) *t* = 60.

Our proposed GPSA can also solve a two-dimensional MMO problem involving a two-dimensional Himmelblau function [33]. Fig 5 shows snapshots of a GPSA simulation run, starting from particles that were randomly placed in the right half-plane (Fig 5(A)). This initial spatial bias was designed to challenge the search for global optima in the left half-plane.

As clarified in Fig 5, the particles again autonomously and dynamically formed multiple sub-swarms. While most of the particles remained in the right half-plane, some escaped to the left half-plane through the repulsive flips. In this run, the first repulsive flip occurred immediately (at *t* = 1) between ***x***_10_(*t*) and ***x***_14_(*t*), causing ***pb***_14_(*t*) to approach one of the distant global optima by *t* = 10 (Fig 5(B)). The ***pb***_23_(*t*) and ***pb***_11_(*t*) then found the remaining distant global optimum by *t* = 60 (Fig 5(C) and 5(D)).

**Fig 6:**
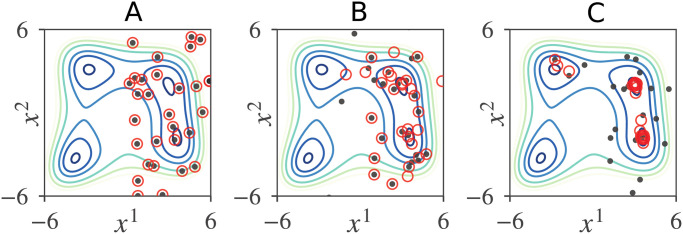
Snapshots of a GPSA run that found only three global optima of the Himmelblau function. (A) *t* = 0. (B) *t* = 10. (C)*t* = 60.

Because our GPSA dynamics include random fluctuations, all global optima are not guaranteed to be found within a finite amount of time (see Fig 6). Nevertheless, as demonstrated in the following sections, the performances of our GPSA and our GPSA with minor improvements were comparable to and considerably superior to those of the existing methods, respectively.

## Performance evaluation

### Experimental setup

This section evaluates our GPSA on the twenty benchmark functions of the CEC 2013 niching-method competition [33]. These benchmark functions were proposed in 2013. However, they are still the latest ones because they have been utilized by the CEC and GECCO niching competitions held between 2013 and 2020. The benchmark functions are listed in Table 2. The terms *d* and *N*^go^ were introduced in (1) and (2), respectively, and *D* is the domain of the function. These benchmark functions are derived from twelve base-test functions, which are broadly classified into two types: functions with local optima and functions with no local optima. The former functions are the Five-Uneven-Peak Trap (*f*_1_), the Uneven Decreasing Maxima (*f*_3_), the Six-Hump Camel Back (*f*_5_), Shubert (*f*_6_, *f*_8_), and the Composite Functions 1 (*f*_11_), 2 (*f*_12_), 3 (*f*_13_, *f*_14_, *f*_16_, *f*_18_), and 4 (*f*_15_, *f*_17_, *f*_19_, *f*_20_). The latter functions include the Equal Maxima (*f*_2_), Himmelblau (*f*_4_), Vincent (*f*_7_, *f*_9_) and the Modified Rastrigin (*f*_10_). These functions over domain *D* are formally defined in [33]. For the external domain of *D*, the value of these functions was set to −10^10^ ≪ *f*_*i*_(*x*), ∀***x*** | ***x*** ∈ *D*, *i* = 1, ⋯, 20 as the penalty value because our GPSA’s particle can exceed the domain *D* due to the repulsive flip.

**Table 2:** Benchmark functions.

*f*	Base-test function	*d*	*N*^go^	*D*	*N*^fes^
*f*_1_	Five-Uneven-Peak Trap	1	2	[0, 30]	5 × 10^4^
*f*_2_	Equal Maxima	1	5	[0, 1]	5 × 10^4^
*f*_3_	Uneven Decreasing Maxima	1	1	[0, 1]	5 × 10^4^
*f*_4_	Himmelblau	2	4	[−6, 6]^2^	5 × 10^4^
*f*_5_	Six-Hump Camel Back	2	2	[−1.9, 1.9] × [−1.1, 1.1]	5 × 10^4^
*f*_6_	Shubert	2	18	[−10, 10]^2^	2 × 10^5^
*f*_7_	Vincent	2	36	[0.25, 10]^2^	2 × 10^5^
*f*_8_	Shubert	3	81	[−10, 10]^3^	4 × 10^5^
*f*_9_	Vincent	3	216	[0.25, 10]^3^	4 × 10^5^
*f*_10_	Modified Rastrigin	2	12	[0, 1]^2^	2 × 10^5^
*f*_11_	Composite Function 1	2	6	[−5, 5]^2^	2 × 10^5^
*f*_12_	Composite Function 2	2	8	[−5, 5]^2^	2 × 10^5^
*f*_13_	Composite Function 3	2	6	[−5, 5]^2^	2 × 10^5^
*f*_14_	Composite Function 3	3	6	[−5, 5]^3^	4 × 10^5^
*f*_15_	Composite Function 4	3	8	[−5, 5]^3^	4 × 10^5^
*f*_16_	Composite Function 3	5	6	[−5, 5]^5^	4 × 10^5^
*f*_17_	Composite Function 4	5	8	[−5, 5]^5^	4 × 10^5^
*f*_18_	Composite Function 3	10	6	[−5, 5]^10^	4 × 10^5^
*f*_19_	Composite Function 4	10	8	[−5, 5]^10^	4 × 10^5^
*f*_20_	Composite Function 4	20	8	[−5, 5]^20^	4 × 10^5^

*d* is the number of dimensions, *N*^go^ is the number of global optima, *D* is the domain of the function, and *N*^fes^ is the number of allowed function evaluations.

Following [33], the number of function evaluations in a single run was set to *N*^fes^ as listed in Table 2. The total number of particles was set to *N* = 500 for *f*_8_ and *f*_9_, which have many global optima, and to *N* = 50 for the remaining functions, as adopted in the existing one-stage methods, RPSO [25] and FERPSO [22]. Each of these particles was updated until *t*_max_ = *N*^fes^/*N*. The number of runs was set to *N*^run^ = 100, at least double the number of runs in previous studies [22, 24, 25, 33].

### Performance metrics

The performance was evaluated by two metrics used in [33]. The first was the peak ratio (PR), which measures the average fraction of the global optima found per run and is given by

$$\begin{array}{c} \text{PR}=\frac{1}{N^{\text{run}}}\sum_{k=1}^{N^{\text{run}}}\frac{n_{k}^{\text{go}}}{N^{\text{go}}}, \end{array}$$ (15)

where $n_{k}^{\text{go}}$ is the number of global optima found during the *k*th run. The $n_{k}^{\text{go}}$ was determined by a standard method provided in [33], which considers that a new global optimum is detected when ***pb***_*i*_(*t*) in (4) satisfies the following two conditions. First, the ***pb***_*i*_(*t*) should be further than any of the already discovered global optima in terms of the distance specified in [33]. Second, the difference between the fitness values of the ***pb***_*i*_(*t*) and the global optima provided in [33] should be less than the accuracy level *ϵ*. Following [33], the accuracy levels in this study were varied as *ϵ* ∈ {10^−1^, 10^−2^, 10^−3^, 10^−4^, 10^−5^}.

The second metric was the success rate (*SR*), which measures the probability of finding all global optima during the same run. The measure is given by

$$\begin{array}{c} \text{SR}=\frac{N^{\text{succ}}}{N^{\text{run}}}, \end{array}$$ (16)

where *N*^succ^ is the number of runs in which all the global optima are found.

The benchmark functions and performance evaluation were implemented in the code provided by the CEC 2013 competition organizers. Obtainable from https://github.com/mikeagn/CEC2013.

### Effects of the parameter *c*_2_

The performance sensitivity of our GPSA to the parameter *c*_2_ was investigated while the other parameters were fixed at *ω* = 0.729 and *c*_1_ = 1.49445.

Table 3 lists the PR-values obtained across all benchmark functions and the accuracy levels for *c*_2_ = 10^−2^, 10^−4^, and 10^−6^, where *D* is the function domain as listed in Table 2. The best values for each function and accuracy-level are highlighted in bold. The bottom section of the table summarizes the averaged *PR* obtained by summing the *PR*-values of all functions and accuracy levels and dividing by the total number of values.

**Table 3:** PR results of our GPSA with different parameters (*c*_2_ = 10^−2^, 10^−4^, and 10^−6^).

	*f*_1_, *D* = [0, 30]			*f*_2_, *D* = [0, 1]			*f*_3_, *D* = [0, 1]			*f*_4_, *D* = [−6, 6]^2^		
*ϵ*	*c*_2_ = 10^−2^,	10^−4^,	10^−6^	*c*_2_ = 10^−2^,	10^−4^,	10^−6^	*c*_2_ = 10^−2^,	10^−4^,	10^−6^	*c*_2_ = 10^−2^,	10^−4^,	10^−6^
10^−1^	0.895	**1.000**	0.995	**1.000**	**1.000**	**1.000**	**1.000**	**1.000**	**1.000**	**1.000**	0.975	0.008
10^−2^	0.210	0.610	**0.970**	**1.000**	**1.000**	**1.000**	**1.000**	**1.000**	**1.000**	**1.000**	0.948	0.005
10^−3^	0.020	0.055	**0.615**	0.996	**1.000**	**1.000**	0.980	**1.000**	**1.000**	0.640	**0.833**	0.003
10^−4^	0.005	0.010	**0.335**	0.812	0.998	**1.000**	0.770	0.990	**1.000**	0.100	**0.583**	0.003
10^−5^	0.000	0.000	**0.190**	0.422	0.894	**0.996**	0.320	0.750	**1.000**	0.018	**0.158**	0.000
	*f*_5_, *D* = [−1.9, 1.9] × [−1.1, 1.1]			*f*_6_, *D* = [−10, 10]^2^			*f*_7_, *D* = [0.25, 10]^2^			*f*_8_, *D* = [−10, 10]^2^		
*ϵ*	*c*_2_ = 10^−2^,	10^−4^,	10^−6^	*c*_2_ = 10^−2^,	10^−4^,	10^−6^	*c*_2_ = 10^−2^,	10^−4^,	10^−6^	*c*_2_ = 10^−2^,	10^−4^,	10^−6^
10^−1^	**1.000**	**1.000**	0.945	**0.173**	0.039	0.003	**0.480**	0.345	0.100	**0.005**	0.000	0.000
10^−2^	**1.000**	**1.000**	0.895	**0.162**	0.036	0.003	**0.480**	0.310	0.049	**0.000**	**0.000**	**0.000**
10^−3^	0.990	**1.000**	0.880	**0.141**	0.027	0.003	**0.480**	0.302	0.036	**0.000**	**0.000**	**0.000**
10^−4^	0.455	**1.000**	0.860	**0.101**	0.018	0.002	**0.467**	0.295	0.032	**0.000**	**0.000**	**0.000**
10^−5^	0.065	0.775	**0.830**	**0.039**	0.016	0.001	**0.309**	0.289	0.031	**0.000**	**0.000**	**0.000**
	*f*_9_, *D* = [0.25, 10]^3^			*f*_10_, *D* = [0, 1]^2^			*f*_11_, *D* = [−5, 5]^2^			*f*_12_, *D* = [−5, 5]^2^		
*ϵ*	*c*_2_ = 10^−2^,	10^−4^,	10^−6^	*c*_2_ = 10^−2^,	10^−4^,	10^−6^	*c*_2_ = 10^−2^,	10^−4^,	10^−6^	*c*_2_ = 10^−2^,	10^−4^,	10^−6^
10^−1^	**0.360**	0.212	0.024	0.981	**0.997**	0.975	**0.667**	0.425	0.017	**0.570**	0.200	0.013
10^−2^	**0.344**	0.139	0.001	0.422	**0.996**	0.975	**0.547**	0.422	0.015	**0.289**	0.195	0.013
10^−3^	**0.219**	0.127	0.000	0.066	0.647	**0.975**	0.128	**0.383**	0.015	0.091	**0.141**	0.009
10^−4^	0.034	**0.114**	0.000	0.008	0.083	**0.933**	0.013	**0.153**	0.012	0.016	**0.071**	0.005
10^−5^	0.001	**0.047**	0.000	0.001	0.006	**0.388**	0.002	**0.028**	0.005	0.000	**0.056**	0.000
	*f*_13_, *D* = [−5, 5]^2^			*f*_14_, *D* = [−5, 5]^3^			*f*_15_, *D* = [−5, 5]^3^			*f*_16_, *D* = [−5, 5]^5^		
*ϵ*	*c*_2_ = 10^−2^,	10^−4^,	10^−6^	*c*_2_ = 10^−2^,	10^−4^,	10^−6^	*c*_2_ = 10^−2^,	10^−4^,	10^−6^	*c*_2_ = 10^−2^,	10^−4^,	10^−6^
10^−1^	**0.657**	0.057	0.003	**0.113**	0.002	0.000	**0.101**	0.000	0.000	**0.003**	0.000	0.000
10^−2^	**0.383**	0.055	0.003	**0.070**	0.002	0.000	**0.059**	0.000	0.000	**0.002**	0.000	0.000
10^−3^	**0.083**	0.050	0.003	**0.052**	0.002	0.000	**0.031**	0.000	0.000	**0.002**	0.000	0.000
10^−4^	0.013	**0.037**	0.003	**0.013**	0.000	0.000	**0.005**	0.000	0.000	**0.000**	**0.000**	**0.000**
10^−5^	0.000	**0.022**	0.003	**0.000**	**0.000**	**0.000**	**0.001**	0.000	0.000	**0.000**	**0.000**	**0.000**
	*f*_17_, *f*_18_, *f*_19_, *f*_20_, *D* = [−5, 5]^*d*^											
*ϵ*	*c*_2_ = 10^−2^,	10^−4^,	10^−6^									
10^−1^	**0.000**	**0.000**	**0.000**	Average								
10^−2^	**0.000**	**0.000**	**0.000**									
10^−3^	**0.000**	**0.000**	**0.000**	*c*_2_ = 10^−2^,	10^−4^,	10^−6^						
10^−4^	**0.000**	**0.000**	**0.000**	0.249	**0.269**	0.222						
10^−5^	**0.000**	**0.000**	**0.000**									

*D* is the function domain as listed in Table 2. The best values for each function and accuracy-level among the *c*_2_ values are indicated in bold.

At the accuracy level *ϵ* = 10^−5^, the PR of the functions with small domains (*f*_1_, *f*_2_, *f*_3_, *f*_5_, and *f*_10_) increased with decreasing *c*_2_, indicating that the exploitation performance of our GPSA improved as *c*_2_ decreased. Note that as the accuracy level *ϵ* decreases, a stricter requirement is imposed on the exploitation performance [39].

In contrast, at the accuracy level *ϵ* = 10^−1^, the PR-values of the large-domain functions (*f*_4_, *f*_6_, *f*_7_, *f*_8_, *f*_9_, and *f*_11_, ⋯, *f*_16_) increased with *c*_2_, indicating that the exploration performance improved as *c*_2_ increased.

The mechanism of these improvements can be explained by the effect of the gravitational force described in the one-dimensional example. Increasing the *c*_2_ increases the distance between the particles generating the repulsive flip ($d\left(t\right)=\sqrt[{3}]{c_{2}}-\epsilon_{+}$ in (14)), elevating the frequency of repulsive flips and reducing that of the attractive motions.

These results clearly demonstrate that the balance between the exploitation and exploration performance of our GPSA can be controlled by *c*_2_; specifically, a large *c*_2_ is suitable for a global searching over a large domain, whereas a small *c*_2_ favors local searching over a small domain.

### Performance comparison with the existing one-stage methods

Next, the performance of our GPSA was compared with those of the existing methods, namely RPSO and FERPSO. These methods were selected for the following reasons. First, they are one-stage methods like our GPSA. Second, they replace the global feedback term (5) of the classical PSO with another term, similarly to our GPSA. Finally, they have the same number of tuning parameters as our GPSA (see Fig 1).

In RPSO, (5) is replaced by

$$\begin{array}{c} a_{i}\left(t\right)={lb}_{i}\left(t\right)-x_{i}\left(t\right), \end{array}$$ (17)

where ${lb}_{i}\left(t\right)\in R^{d}$ is the local best of the *i*th particle at iteration *t*. The local best is the highest-cost position found by the particle or one of its neighbors, i.e., the best among the personal bests ***pb***_*i*−1_(*t*), ***pb***_*i*_(*t*), and ***pb***_*i*+1_(*t*).

In FERPSO, (5) is replaced with:

$$\begin{array}{c} a_{i}\left(t\right)={nb}_{i}\left(t\right)-x_{i}\left(t\right), \end{array}$$ (18)

where ${nb}_{i}\left(t\right)\in R^{d}$ again represents a neighborhood personal best, but selected by maximizing the Euclidean-distance ratio (FER):

$$\begin{array}{c} {\text{FER}}_{ji}=\alpha \cdot \frac{f\left({pb}_{j}\left(t\right)\right)-f\left({pb}_{i}\left(t\right)\right)}{\left|\right|{pb}_{j}\left(t\right)-{pb}_{i}\left(t\right)\left|\right|}, \end{array}$$ (19)

where *α* = ||*s*||/(*f*(***gb***(*t*)) − *f*(***x***_*w*_(*t*))) is a scaling factor, ||*s*|| is the size of the search space [22], ***x***_*w*_(*t*) is the least-fitted particle in the current population, and ***pb***_*i*_(*t*) and ***pb***_*j*_(*t*) are the personal bests of the *i*th and *j*th particles, respectively.

In this study, the tuning parameters of RPSO and FERPSO were set to *ω* = 0.729 and *c*_1_ = *c*_2_ = 1.49445 consistent with [22, 25]. The other parameters and experimental conditions were consistent with those in the previous section.

Table 4 lists the *SR*-values in (16) obtained by our GPSA and the compared methods. The best and averaged values are indicated and summarized, as described in Table 3.

**Table 4:** SR results of our GPSA with *c*_2_ = 10^−2^, 10^−4^, and 10^−6^, RPSO, and FERPSO.

	*f*_1_, *D* = [0, 30]					*f*_2_, *D* = [0, 1]					*f*_3_, *D* = [0, 1]				
	GPSA			RPSO	FERPSO	GPSA			RPSO	FERPSO	GPSA			RPSO	FERPSO
*ϵ*	*c*_2_ = 10^−2^,	10^−4^,	10^−6^			*c*_2_ = 10^−2^,	10^−4^,	10^−6^			*c*_2_ = 10^−2^,	10^−4^,	10^−6^		
10^−1^	0.80	**1.00**	0.99	**1.00**	**1.00**	**1.00**	**1.00**	**1.00**	0.84	0.90	**1.00**	**1.00**	**1.00**	**1.00**	**1.00**
10^−2^	0.03	0.32	0.94	**1.00**	**1.00**	**1.00**	**1.00**	**1.00**	0.84	0.90	**1.00**	**1.00**	**1.00**	**1.00**	**1.00**
10^−3^	0.00	0.01	0.39	**1.00**	**1.00**	0.98	**1.00**	**1.00**	0.84	0.90	0.98	**1.00**	**1.00**	**1.00**	**1.00**
10^−4^	0.00	0.00	0.13	**1.00**	**1.00**	0.29	0.99	**1.00**	0.84	0.90	0.77	0.99	**1.00**	**1.00**	**1.00**
10^−5^	0.00	0.00	0.03	**1.00**	**1.00**	0.01	0.52	**0.98**	0.79	0.89	0.32	0.75	**1.00**	**1.00**	**1.00**
	*f*_4_, *D* = [−6, 6]^2^					*f*_5_, *D* = [−1.9, 1.9] × [−1.1, 1.1]					*f*_10_, *D* = [0, 1]^2^				
	GPSA			RPSO	FERPSO	GPSA			RPSO	FERPSO	GPSA			RPSO	FERPSO
*ϵ*	*c*_2_ = 10^−2^,	10^−4^,	10^−6^			*c*_2_ = 10^−2^,	10^−4^,	10^−6^			*c*_2_ = 10^−2^,	10^−4^,	10^−6^		
10^−1^	**1.00**	0.92	0.00	0.94	0.87	**1.00**	**1.00**	0.89	**1.00**	**1.00**	0.79	**0.96**	0.71	0.00	0.00
10^−2^	**1.00**	0.84	0.00	0.77	0.87	**1.00**	**1.00**	0.79	**1.00**	**1.00**	0.00	**0.95**	0.71	0.00	0.00
10^−3^	0.18	0.46	0.00	0.63	**0.87**	0.98	**1.00**	0.77	**1.00**	**1.00**	0.00	0.00	**0.71**	0.00	0.00
10^−4^	0.00	0.09	0.00	0.61	**0.87**	0.21	**1.00**	0.74	**1.00**	**1.00**	0.00	0.00	**0.42**	0.00	0.00
10^−5^	0.00	0.00	0.00	0.58	**0.87**	0.00	0.58	0.69	**1.00**	**1.00**	**0.00**	**0.00**	**0.00**	**0.00**	**0.00**
	*f*_6_, *f*_7_, *f*_8_, *f*_9_, *f*_11_, ⋯, *f*_20_														
	GPSA			RPSO	FERPSO										
*ϵ*	*c*_2_ = 10^−2^,	10^−4^,	10^−6^												
10^−1^	**0.00**	**0.00**	**0.00**	**0.00**	**0.00**	Average									
10^−2^	**0.00**	**0.00**	**0.00**	**0.00**	**0.00**	GPSA			RPSO	FERPSO					
10^−3^	**0.00**	**0.00**	**0.00**	**0.00**	**0.00**	*c*_2_ = 10^−2^,	10^−4^,	10^−6^							
10^−4^	**0.00**	**0.00**	**0.00**	**0.00**	**0.00**	0.14	0.19	0.19	0.23	**0.24**					
10^−5^	**0.00**	**0.00**	**0.00**	**0.00**	**0.00**										

*D* is the function domain, as listed in Table 2. The best values for each function and accuracy-level among RPSO, FERPSO and our GPSA with *c*_2_ = 10^−2^, 10^−4^, and 10^−6^ are highlighted in bold.

On the small-domain function *f*_10_, our GPSA obtained remarkably higher *SR*-values than the compared methods. In particular, for *ϵ* = 10^−1^, 10^−2^, 10^−3^ and 10^−4^ and *c*_2_ = 10^−6^, the *SR*-value of our GPSA exceeded 0.4, versus *SR* = 0 in the compared methods. On the other small-domain functions *f*_2_ and *f*_3_, the *SR*-values of our GPSA with *c*_2_ = 10^−6^ either outperformed or equaled those of the compared methods. These results showed that when *c*_2_ is relatively small, the exploitation performance of our GPSA is comparable to those of other methods.

On the large-domain functions, *f*_4_ and *f*_5_ with *ϵ* = 10^−1^ and 10^−2^ and *c*_2_ = 10^−2^, our GPSA performed at least as well as the compared methods. These results indicate that when *c*_2_ is a relatively large, the exploration performance of our GPSA is comparable to those of other methods.

However, in terms of the averaged *SR* values, the other methods outperformed our GPSA. This degradation was primarily attributed to the dilemma of choosing between the exploitation and exploration performance in our GPSA.

## Improvement of GPSA with a dynamic *c*_2_

To resolve the exploration—exploitation dilemma of our GPSA, this section introduces a dynamic *c*_2_ that further improves its performance.

### Dynamic *c*_2_ for our GPSA

**Fig 7:**
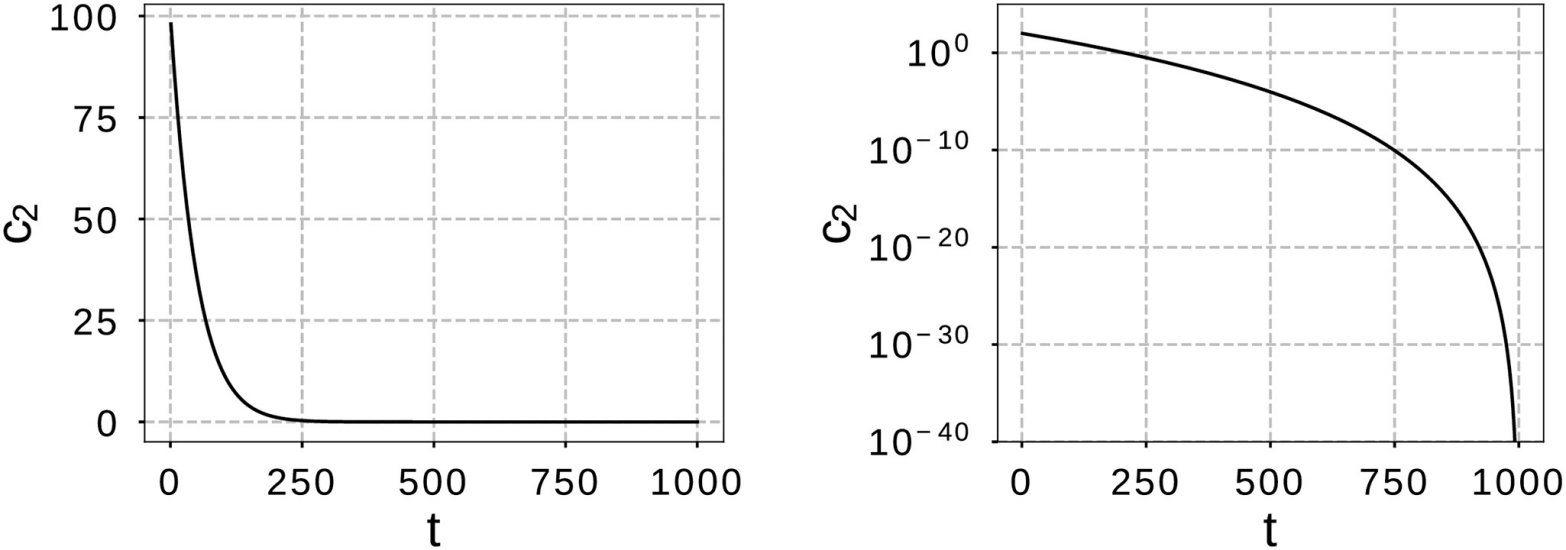
Dynamic *c*_2_ as a function of iteration *t*, for $c_{2}^{\text{ini}}=10^{2}$, *n* = 20, and *t*_max_ = 1000.

Given our observations in the previous section, it is inferred that our GPSA’s iteration should initially start with a large *c*_2_ to enhance the exploration performance, and that *c*_2_ should decrease as the iteration increments to improve the exploitation performance. Therefore, the present study proposes a method with a dynamic *c*_2_, in which *c*_2_ is provided as a nonlinear function of the iteration *t* based on the dynamic *ω* as used successfully in the classical PSOs [21, 40, 41], as follows:

$$\begin{array}{c} c_{2}\left(t\right)=c_{2}^{\text{ini}}\{\frac{{\left(t_{\max}-t\right)}^{n}}{{\left(t_{\max}\right)}^{n}}\}, \end{array}$$ (20)

where $c_{2}^{\text{ini}}$ is the initial *c*_2_ at the beginning of a run and *n* is the nonlinear modulation index. In this study, these parameters were set to $c_{2}^{\text{ini}}=10^{2}$ and *n* = 20 to cover the *c*_2_ range analyzed in the previous section. Fig 7 shows the dynamic *c*_2_ as a function of *t* for *t*_max_ = 1000, where the left graph is a linear plot and the right a semi-log plot. It is shown that the *c*_2_ gradually decreased to zero as *t* increased, and *c*_2_ used in Table 3 was covered. In this study, this modified GPSA is called a dynamic GPSA (DGPSA).

Our DGPSA proposed above is more complicated than our GPSA, however, it is still simpler than the existing one-stage methods in Fig 1, except for RPSO (FERPSO, LIPS, SPSO and NGSA), the reason being that the computational complexity of our DGPSA is *O*(*N*^2^ + 1) obtained by summing the complexities *O*(*N*^2^) of original GPSA and *O*(1) of (20), and is even lower than FERPSO, LIPS, SPSO, and NGSA. In addition, the number of tuning parameters of our DGPSA, $\left(\omega ,c_{1},c_{2}^{\text{ini}},n\right)\in R^{4}$, is fewer than or equivalent to that of LIPS, SPSO and NGSA.

### Performance of our DGPSA

Table 5 shows the *PR*-values obtained by our DGPSA and the compared methods.

**Table 5:** PR results of our DGPSA, RPSO, and FERPSO.

	*f*_1_			*f*_2_			*f*_3_			*f*_4_		
*ϵ*	DGPSA	RPSO	FERPSO	DGPSA	RPSO	FERPSO	DGPSA	RPSO	FERPSO	DGPSA	RPSO	FERPSO
10^−1^	**1.000**	**1.000**	**1.000**	**1.000**	0.968	0.980	**1.000**	**1.000**	**1.000**	**1.000**	0.985	0.968
10^−2^	**1.000**	**1.000**	**1.000**	**1.000**	0.968	0.980	**1.000**	**1.000**	**1.000**	**1.000**	0.940	0.968
10^−3^	**1.000**	**1.000**	**1.000**	**1.000**	0.968	0.980	**1.000**	**1.000**	**1.000**	**1.000**	0.903	0.968
10^−4^	**1.000**	**1.000**	**1.000**	**1.000**	0.968	0.980	**1.000**	**1.000**	**1.000**	**1.000**	0.895	0.968
10^−5^	**1.000**	**1.000**	**1.000**	**1.000**	0.958	0.978	**1.000**	**1.000**	**1.000**	**1.000**	0.885	0.968
	*f*_5_			*f*_6_			*f*_7_			*f*_8_		
*ϵ*	DGPSA	RPSO	FERPSO	DGPSA	RPSO	FERPSO	DGPSA	RPSO	FERPSO	DGPSA	RPSO	FERPSO
10^−1^	**1.000**	**1.000**	**1.000**	**0.948**	0.486	0.409	**0.430**	0.194	0.195	0.594	**0.607**	0.374
10^−2^	**1.000**	**1.000**	**1.000**	**0.948**	0.468	0.402	**0.430**	0.194	0.195	0.586	**0.591**	0.360
10^−3^	**1.000**	**1.000**	**1.000**	**0.948**	0.449	0.398	**0.430**	0.191	0.195	0.575	**0.580**	0.345
10^−4^	**1.000**	**1.000**	**1.000**	**0.948**	0.436	0.394	**0.427**	0.182	0.194	0.561	**0.571**	0.331
10^−5^	**1.000**	**1.000**	**1.000**	**0.948**	0.426	0.394	**0.415**	0.175	0.194	0.531	**0.565**	0.317
	*f*_9_			*f*_10_			*f*_11_			*f*_12_		
*ϵ*	DGPSA	RPSO	FERPSO	DGPSA	RPSO	FERPSO	DGPSA	RPSO	FERPSO	DGPSA	RPSO	FERPSO
10^−1^	**0.313**	0.213	0.179	**0.990**	0.677	0.569	**0.667**	0.598	0.633	**0.728**	0.360	0.500
10^−2^	**0.257**	0.191	0.179	**0.990**	0.666	0.569	**0.667**	0.588	0.633	**0.718**	0.353	0.499
10^−3^	**0.212**	0.168	0.177	**0.990**	0.648	0.568	**0.667**	0.580	0.633	**0.708**	0.341	0.496
10^−4^	**0.193**	0.153	0.174	**0.990**	0.636	0.568	**0.667**	0.578	0.633	**0.699**	0.334	0.496
10^−5^	**0.186**	0.140	0.164	**0.990**	0.618	0.564	**0.667**	0.575	0.633	**0.694**	0.333	0.496
	*f*_13_			*f*_14_			*f*_15_			*f*_16_		
*ϵ*	DGPSA	RPSO	FERPSO	DGPSA	RPSO	FERPSO	DGPSA	RPSO	FERPSO	DGPSA	RPSO	FERPSO
10^−1^	**0.660**	0.533	0.548	**0.658**	0.553	0.528	**0.343**	0.288	0.289	**0.577**	0.537	0.322
10^−2^	**0.660**	0.522	0.548	**0.658**	0.547	0.528	**0.341**	0.284	0.289	**0.570**	0.530	0.322
10^−3^	**0.660**	0.510	0.548	**0.658**	0.543	0.528	**0.329**	0.281	0.288	**0.570**	0.527	0.322
10^−4^	**0.660**	0.505	0.548	**0.657**	0.540	0.528	**0.321**	0.280	0.288	**0.570**	0.520	0.322
10^−5^	**0.660**	0.505	0.548	**0.645**	0.538	0.528	**0.316**	0.279	0.288	**0.570**	0.517	0.322
	*f*_17_			*f*_18_			*f*_19_			*f*_20_		
*ϵ*	DGPSA	RPSO	FERPSO	DGPSA	RPSO	FERPSO	DGPSA	RPSO	FERPSO	DGPSA	RPSO	FERPSO
10^−1^	0.219	**0.244**	0.125	0.167	**0.250**	0.177	**0.125**	0.114	0.036	0.125	**0.134**	0.090
10^−2^	0.211	**0.241**	0.125	0.167	**0.247**	0.177	**0.125**	0.114	0.036	0.125	**0.134**	0.090
10^−3^	0.206	**0.240**	0.125	0.167	**0.247**	0.175	**0.125**	0.114	0.036	0.125	**0.134**	0.090
10^−4^	0.206	**0.240**	0.125	0.167	**0.247**	0.175	**0.125**	0.114	0.036	0.125	**0.134**	0.090
10^−5^	0.206	**0.239**	0.124	0.167	**0.247**	0.175	**0.125**	0.114	0.036	0.125	**0.134**	0.090
	Average											
	DGPSA	RPSO	FERPSO									
	**0.619**	0.523	0.494									

The best values for each function and accuracy level among the algorithms are indicated in bold.

First, these values of our DGPSA were compared with our GPSA results in Table 3. On *f*_6_, *f*_8_, and *f*_12_, ⋯, *f*_20_, our DGPSA obtained higher *PR*-values at all accuracy levels than our GPSA results. Even on *f*_1_, *f*_2_, *f*_4_, *f*_5_, *f*_7_, *f*_9_, *f*_10_, and *f*_11_, our DGPSA also had higher *PR*-values at the strictest accuracy level *ϵ* = 10^−5^. Furthermore, the averaged *PR*-value increased by more than 2.3 times. These results clearly demonstrate that our DGPSA successfully solves the dilemma found in our GPSA.

Furthermore, our DGPSA obtained higher PR-values than the compared methods on *f*_2_, *f*_4_, *f*_6_, *f*_7_, *f*_9_, ⋯, *f*_16_, and *f*_19_. In particular, on *f*_6_ and *f*_7_, the *PR*-values of our DGPSA were at least 1.9 times higher than those of the compared methods. The averaged *PR*-values also indicated the superiority of our DGPSA.

Table 6 shows the SR-values obtained by our DGPSA, all of which were superior or equal to those of the compared methods shown in Table 4.

**Table 6:** SR results of our DGPSA for all benchmark functions and all accuracy levels.

*ϵ*	*f*_1_	*f*_2_	*f*_3_	*f*_4_	*f*_5_	*f*_6_	*f*_10_	*f*_12_	*f*_7_, *f*_8_, *f*_9_, *f*_11_, *f*_13_, ⋯, *f*_20_	
10^−1^	1.000	1.000	1.000	1.000	1.000	0.320	0.880	0.030	0.000	Average
10^−2^	1.000	1.000	1.000	1.000	1.000	0.320	0.880	0.030	0.000	
10^−3^	1.000	1.000	1.000	1.000	1.000	0.320	0.880	0.020	0.000	0.311
10^−4^	1.000	1.000	1.000	1.000	1.000	0.320	0.880	0.020	0.000	
10^−5^	1.000	1.000	1.000	1.000	1.000	0.320	0.880	0.020	0.000	

The results above show that our DGPSA outperformed the compared methods in terms of *PR* and *SR*.

### Statistical tests

The statistically significant tests were not yet conducted in the results shown above. Here, the number of functions is counted for which our DGPSA significantly outperformed the others, as was done in [39].

Consider the following sets:

$$\begin{array}{c} N_{i}(\epsilon )≔\{n_{k}^{\text{go}}\left(f_{i},\epsilon \right)\;|\;1\leq k\leq 100\},\; \end{array}$$ (21)

$$\begin{array}{c} {\bar{N}}_{i}(\epsilon )≔\{\bar{n_{k}^{\text{go}}}\left(f_{i},\epsilon \right)\;|\;1\leq k\leq 100\}, \end{array}$$ (22)

where $n_{k}^{\text{go}}\left(f_{i},\epsilon \right)$ is $n_{k}^{\text{go}}$ in (15) obtained by our DGPSA on the function *f*_*i*_ (*i* = 1, ⋯, 20) and the accuracy level *ϵ*, $N_{i}(\epsilon )$ is a set of the $n_{k}^{\text{go}}\left(f_{i},\epsilon \right)$ for all *k*, and $\bar{n_{k}^{\text{go}}}\left(f_{i},\epsilon \right)$ and ${\bar{N}}_{i}(\epsilon )$ are those of the compared method. Then, to classify the *f*_*i*_, the following sets were introduced:

$$\begin{array}{c} F^{+}\left(\epsilon \right)≔\{f_{i}\;|\;m_{i}>{\bar{m}}_{i},\;\;\text{and}\;\;T_{i}=1\}, \end{array}$$ (23)

$$\begin{array}{c} F^{-}\left(\epsilon \right)≔\{f_{i}\;|\;m_{i}<{\bar{m}}_{i},\;\;\text{and}\;\;T_{i}=1\}, \end{array}$$ (24)

$$\begin{array}{c} F^{0}\left(\epsilon \right)≔\{f_{i}\;|\;T_{i}=0\}. \end{array}$$ (25)

Here, *m*_*i*_ and ${\bar{m}}_{i}$ indicate the median of $N_{i}(\epsilon )$ and ${\bar{N}}_{i}(\epsilon )$, respectively.*T*_*i*_ becomes unity when there is a significant difference between *m*_*i*_ and ${\bar{m}}_{i}$, and otherwise zero. This significant difference was determined by the Wilcoxon rank-sum test [42] with a significance level of 0.05, as used in [39]. $F^{+}\left(\epsilon \right)$ and $F^{-}\left(\epsilon \right)$ are the sets of *f*_*i*_ on which our DGPSA was significantly superior and inferior to the compared method, respectively. Also, $F^{0}\left(\epsilon \right)$ is the set of *f*_*i*_ where there was no significant difference between our DGPSA and the compared method.

Table 7 shows the resulting numbers of functions $\#F^{+}\left(\epsilon \right)$, $\#F^{-}\left(\epsilon \right)$, and $\#F^{0}\left(\epsilon \right)$, where #*A* denotes the number of elements of a set *A*. Here, Sum(*ϵ*) indicates the sum of $\#F^{+}\left(\epsilon \right)$, $\#F^{-}\left(\epsilon \right)$, and $\#F^{0}\left(\epsilon \right)$. The values in parentheses are the composition ratios of the resulting number to the Sum(*ϵ*).

**Table 7:** Resulting numbers of functions in $F^{+}\left(\epsilon \right)$, $F^{-}\left(\epsilon \right)$, and $F^{0}\left(\epsilon \right)$.

	RPSO versus DGPSA				
	*ϵ* = 10^−1^	*ϵ* = 10^−2^	*ϵ* = 10^−3^	*ϵ* = 10^−4^	*ϵ* = 10^−5^
$\#F^{+}\left(\epsilon \right)$	12 (60%)	12 (60%)	12 (60%)	12 (60%)	12 (60%)
$\#F^{-}\left(\epsilon \right)$	4 (20%)	3 (15%)	3 (15%)	4 (20%)	4 (20%)
$\#F^{0}\left(\epsilon \right)$	4 (20%)	5 (25%)	5 (25%)	4 (20%)	4 (20%)
Sum(*ϵ*)	20 (100%)	20 (100%)	20 (100%)	20 (100%)	20 (100%)
	FERPSO versus DGPSA				
	*ϵ* = 10^−1^	*ϵ* = 10^−2^	*ϵ* = 10^−3^	*ϵ* = 10^−4^	*ϵ* = 10^−5^
$\#F^{+}\left(\epsilon \right)$	16 (80%)	16 (80%)	16 (80%)	16 (80%)	16 (80%)
$\#F^{-}\left(\epsilon \right)$	1 (5%)	1 (5%)	1 (5%)	1 (5%)	1 (5%)
$\#F^{0}\left(\epsilon \right)$	3 (15%)	3 (15%)	3 (15%)	3 (15%)	3 (15%)
Sum(*ϵ*)	20 (100%)	20 (100%)	20 (100%)	20 (100%)	20 (100%)

#*A* denotes the number of elements of a set *A*, Sum(*ϵ*) indicates the sum of $\#F^{+}\left(\epsilon \right)$, $\#F^{-}\left(\epsilon \right)$, and $\#F^{0}\left(\epsilon \right)$, and the values in the parentheses indicate the composition ratios of the resulting number to the Sum(*ϵ*).

Focusing on the comparison between RPSO and our DGPSA shown in the top of Table 7, it is understood that $\#F^{+}\left(\epsilon \right)$ were equal to 12 for all *ϵ*, and that their composition ratios were 60% $\left(=\#F^{+}\left(\epsilon \right)/\text{Sum}\left(\epsilon \right)\times 100\;\%\right)$. From the definition of (23), these results show that our DGPSA was significantly superior to RPSO for 60% of the benchmark functions. On the other hand, $\#F^{-}\left(\epsilon \right)$ were equal to or less than 4, and their composition ratios were 20% or less. From the definition of (24), these results show that our DGPSA was significantly inferior to RPSO in at most 20% of the benchmark functions.

Next, focusing on the comparison between FERPSO and our DGPSA shown in the bottom of the table, it is shown that $\#F^{+}\left(\epsilon \right)$ were equal to 16 and that their composition ratios were 80%, as well as that $\#F^{-}\left(\epsilon \right)$ were equal to 1 and their composition ratios were 5%. This indicates that our DGPSA was significantly superior and inferior to FERPSO for 80% and 5% of the benchmark functions, respectively.

Therefore, these results clearly demonstrate that our DGPSA was significantly superior to the compared one-stage methods in at least 60% of the benchmark functions.

### Runtime comparison

**Fig 8:**
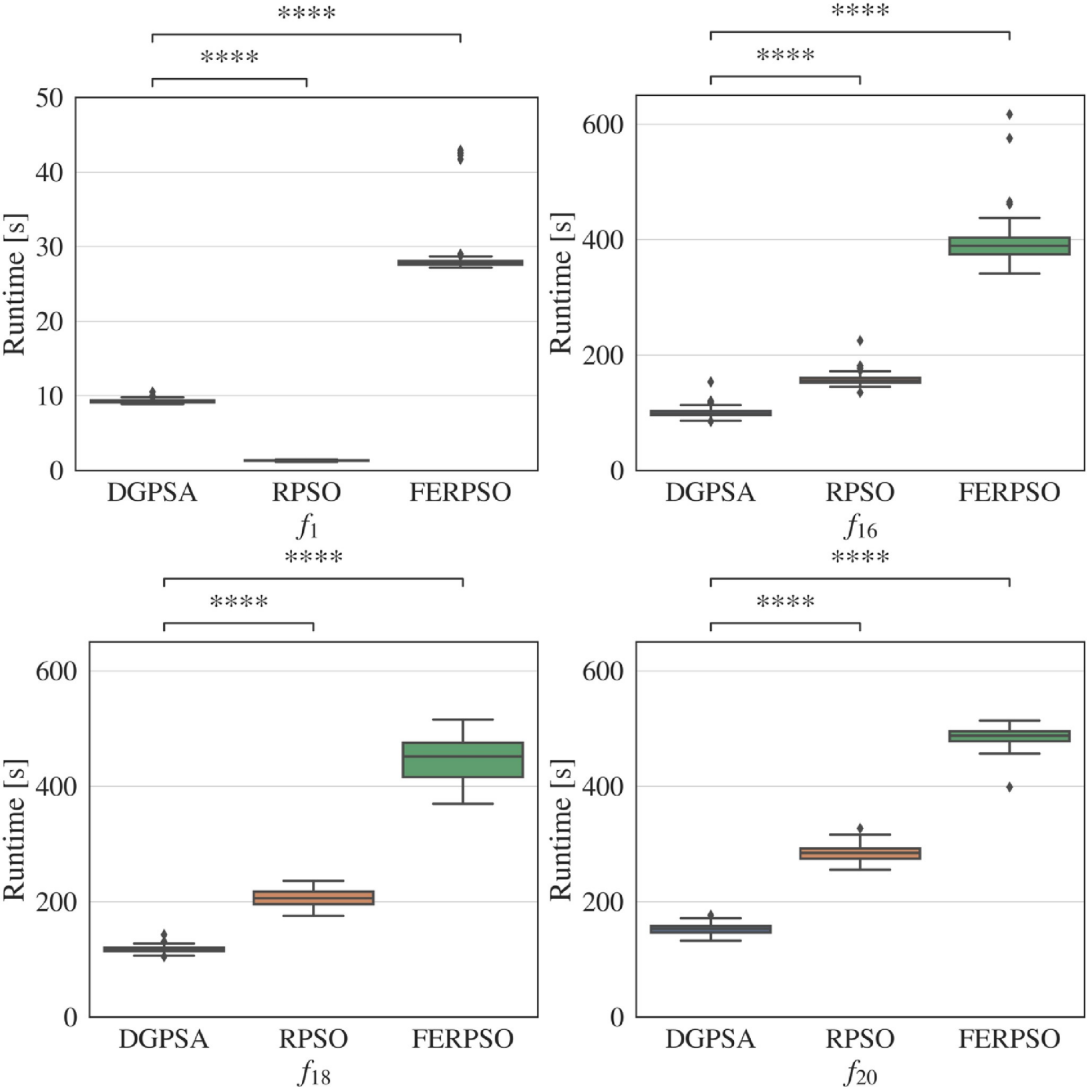
Comparison of runtime of our DGPSA and existing one-stage methods, where **** indicates significantly difference between the runtime of our DGPSA and that of the compered method with *p*-value <10^−4^.

Fig 8 shows the boxplot of runtime performed by our DGPSA, RPSO and FERPSO for one-, five-, ten-, and twenty-dimensional functions: *f*_1_, *f*_16_, *f*_18_, *f*_20_. The simulation conditions were consistent with those of the performance comparisons in the previous sub section. In Fig 8, **** indicates the statistically significant difference with *p*-value <10^−4^ between our DGPSA and the compered method, which were tested by Wilcoxon rank-sum test [42]. All algorithms were implemented by Python and executed by the same physical computer (Lenovo ThinkPad T480s, Intel Core i5 processor with 24GB Memory).

Focusing on the runtime comparison between our DGPSA and FERPSO, the runtime of our DGPSA was statistically significantly shorter than that of FERPSO. The mechanism of these results can be explained by the computational complexity described in Fig 1. Focusing on the comparison between our DGPSA and RPSO, our DGPSA was considerably inferior to RPSO for the one-dimensional function *f*_1_. On the other hand, for the high-dimensional functions *f*_16_, *f*_18_, and *f*_20_, our DGPSA showed a statistically significantly shorter runtime than those of RPSO.

These results demonstrated that although our DGPSA has higher computational complexity than RPSO (see Fig 1), its execution time was significantly shorter than RPSO for the high-dimensional functions.

### Performance comparison with the existing two-stage methods

The performance of our DGPSA was also compared with those of the existing two-stage methods, namely, RS-CMSA and HillVallEA19, that are the winner of the competition on niching methods held in GECCO 2017 and 2019. Table 8 compares the *PR*-values of our DGPSA for *ϵ* = 10^−5^ (reproduction from Table 5) with those of the two-stage methods in [29, 43], where the benchmark problems and simulation conditions were consistent with those of this study. Table 8 also shows the statistical test results between our DGPSA and the existing two-stage methods, which was conducted similar to the previous section (Statistical test). The original results of RS-CMSA and HillVallEA19 were obtained from the repository provided by the competition organizers https://github.com/mikeagn/CEC2013. The symbol (0) indicates that there was no statistically significant difference between our DGPSA and the compared method. The symbol (−) indicates that our DGPSA was significantly inferior to the compared method with a significance level of 0.05.

**Table 8:** *PR* results of our DGPSA and the existing two-stage methods with *ϵ* = 10^−5^.

*f*	DGPSA	RS-CMSA	HillVallEA19	*f*	DGPSA	RS-CMSA	HillVallEA19
*f*_1_	1.000	1.000 (0)	1.000 (0)	*f*_11_	0.667	0.997 (-)	1.000 (-)
*f*_2_	1.000	1.000 (0)	1.000 (0)	*f*_12_	0.694	0.948 (-)	1.000 (-)
*f*_3_	1.000	1.000 (0)	1.000 (0)	*f*_13_	0.660	0.997 (-)	1.000 (-)
*f*_4_	1.000	1.000 (0)	1.000 (0)	*f*_14_	0.645	0.810 (-)	0.917 (-)
*f*_5_	1.000	1.000 (0)	1.000 (0)	*f*_15_	0.316	0.748 (-)	0.750 (-)
*f*_6_	0.948	0.999 (-)	1.000 (-)	*f*_16_	0.570	0.667 (-)	0.687 (-)
*f*_7_	0.415	0.997 (-)	1.000 (-)	*f*_17_	0.206	0.703 (-)	0.750 (-)
*f*_8_	0.531	0.871 (-)	0.975 (-)	*f*_18_	0.167	0.667 (-)	0.667 (-)
*f*_9_	0.186	0.730 (-)	0.972 (-)	*f*_19_	0.125	0.503 (-)	0.585 (-)
*f*_10_	0.990	1.000 (-)	1.000 (-)	*f*_20_	0.125	0.483 (-)	0.482 (-)
				average	0.612	0.856	0.892

The symbol (0) indicates that there was no statistically significant difference between our DGPSA and the compared method and (−) indicates that our DGPSA was significantly inferior to the compared method, with a significance level of 0.05.

Our DGPSA performed comparably to the existing two-stage methods on functions *f*_1_, *f*_2_, *f*_3_, *f*_4_, and *f*_5_ with no statistical difference. Although the *PR*-values for the other functions were significantly inferior in our DGPSA than in the two-stage methods, our one-stage DGPSA is suitable for non-experts in optimization algorithms who wish to implement an MMO algorithm into the production items (as described in GPSA’s section).

## Conclusions

This study proposed a new MMO algorithm called GPSA, which replaces the global feedback term in the classical PSO by an inverse-square gravitational force term between the particles, in order to resolve the drawbacks of existing MMO algorithms, namely, the difficulties in understanding and implementing two-stage methods, and the high computational complexity, large number of tuning parameters and the limited performance in one-stage methods. The proposed GPSA is a simple and purely dynamical algorithm, which is distinct from the existing two- and one-stage MMO methods because of absence of clustering algorithms, restart schemes, taboo archives, and algorithmic procedures for selecting the social and nearest best(s).

First, the types of niching behavior generated by our GPSA were investigated on simple one- and two-dimensional MMO problems. The findings are summarized below:

- Through mutual attraction via the inverse-square gravitational force, the particles dynamically formed into sub-swarms dynamically without algorithmic rules.
- The sub-swarms autonomously gathered near the multiple global optima, with individual particles performing damped oscillations about their respective personal bests.
- A few of the particles in the sub-swarms intermittently escaped, via the repulsive flip, and found distant global optima.

Next, our GPSA was compared with the existing MMO algorithms. The observations are summarized below:

- In the nominal performance comparison, our GPSA was confirmed as a simpler method than the existing methods, because it is less computationally complex and requires fewer tuning parameters than the existing high-performance methods.
- In the actual performance comparison, *PR* and *SR* were measured on the twenty CEC benchmark functions. The exploitation and exploration performances of our GPSA were comparable to those of the existing one-stage methods (RPSO and FERPSO).

Finally, an improved GPSA called DGPSA, which gradually decreases the parameter *c*_2_ as the iterations proceed, was evaluated and the results are summarized below:

- Although our DGPSA is simpler than the existing one-stage methods, it outperformed the compared one-stage methods in both *PR* and *SR*.
- The well-known statistical test method, namely, the Wilcoxon rank-sum test, confirmed the superiority of our DGPSA over the compared one-stage methods on at least 60% of the benchmark functions.
- In comparison of runtime for high-dimensional functions, our DGPSA was significantly superior to the compared one-stage methods.
- Although our DGPSA was statistically inferior to the existing two-stage methods for high-dimensional functions, its simplicity enables its implementation as an MMO algorithm by non-experts.

Clearly, our proposed DGPSA resolves the shortcomings of the existing methods by virtue of its simple and purely dynamical algorithm that outperforms the existing one-stage methods (FERPSO and RPSO). Therefore, we believe that the proposed DGPSA is a more appropriate algorithm than the existing methods for the situation where non-experts in optimization algorithms understand and implement a MMO algorithm to solve the real world problems.

In the future, we plan to investigate the applicability of our GPSA to real-world optimization problems, including optimal structure design [6, 11] and the training of neural networks. We will also consider ways of optimizing the model parameters and applying dynamic inertia weight.
